# Healthcare professionals’ knowledge and perceptions of infant formulas composition: a comparative study of Spanish pediatricians and pharmacy professionals

**DOI:** 10.3389/fnut.2026.1801406

**Published:** 2026-07-08

**Authors:** Luis Ortiz-González, Alicia Santamaría-Orleans, Óscar Llansó-Sánchez, Paula Fernández-Ribal, Maite Pérez-Hernández, Cristóbal Coronel-Rodríguez

**Affiliations:** 1 Department of Biomedical Sciences, Medicine and Health Sciences Faculty, Universidad de Extremadura, Badajoz, Spain; 2 Scientific Communication, Laboratorios Ordesa SL, Barcelona, Spain; 3 Food and Nutrition Area, Barcelona Official College of Pharmacists, Barcelona, Spain; 4 Central Pharmacy, Gelida, Barcelona, Spain; 5 Medical Department, Laboratorios Ordesa SL, Barcelona, Spain; 6 Department of Pharmacology, Pediatrics and Radiology, Faculty of Medicine, Amante Laffón Health Care Centre, Sevilla, Spain

**Keywords:** functional ingredients, HCPs self-perceived knowledge, infant formula, nutritional knowledge, parental education

## Abstract

**Introduction:**

As infant formulas evolve to include bioactive components that mimic breast milk, the knowledge and perceptions of healthcare professionals (HCPs) about these innovations become critical. Pediatricians and pharmacy professionals play complementary roles in recommending and guiding infant formula use. However, disparities in understanding and communication may impact caregiver education and infant health outcomes. This study aims to assess the perceptions of Spanish pediatricians and pharmacy professionals regarding infant formulas, focusing on the recognition of ingredients, the importance attributed to them, and the understanding of their biological functions. Differences between the two professional groups were analyzed to identify gaps and educational needs.

**Methods:**

An observational, cross-sectional survey was conducted with 876 responding Spanish pediatricians (*n* = 505) and pharmacy professionals (*n* = 371) via anonymous online questionnaires and e-mails. The survey comprised 28 questions across three sections: sociodemographic, general perceptions of infant formulas, and functional recognition of 12 specific ingredients. Quantitative and categorical data were analyzed using appropriate statistical tests, with comparisons made between the two groups.

**Results:**

Surveyed pediatricians reported higher self-perceived knowledge than pharmacy professionals (mean score 7.9 vs. 6.7, *p* < 0.0001) and showed significantly better questionnaire-based performance in identifying at least one relevant biological function of infant formula ingredients. However, surveyed pharmacy professionals placed greater emphasis on the need to educate parents (*p* < 0.0001). Notable gaps emerged between self-perceived knowledge and questionnaire-based functional recognition. Among participants who reported knowing certain ingredients “very well,” some were unable to correctly identify at least one relevant biological function. Both groups primarily relied on pharmaceutical industry materials for information (85.0% of pediatricians and 84.4% of pharmacy professionals).

**Conclusion:**

Responding pediatricians and pharmacy professionals showed differences in their functional recognition and perception of infant formula ingredients. While the former excelled in the questionnaire-based technical recognition, the latter did so in parental education. Both profiles provide complementary strengths and show opportunities to improve knowledge about some bioactive ingredients. These results suggest potential opportunities for specific ongoing training and collaborative approaches that may help ensure consistent, evidence-based advice for caregivers.

## 1 Introduction

Breast milk is recognized as the optimal nutrition for infants. Its unique composition contributes to the growth and development of the infant’s digestive, immune, and neurological systems (1, 2), while breastfeeding is associated with reduced risk of infections, allergies and chronic diseases later in life (3, 4). International public health guidelines recommend that infants should be exclusively breastfed for the initial 6 months of life to ensure optimal growth, development, and overall health. Following this period, complementary foods should be introduced while breastfeeding should ideally continue up to 2 years of age or beyond (5, 6). However, mother’s decision to breastfeed is purely personal and can be influenced by multiple factors. In addition, under certain situations, breastfeeding might not be possible or sufficient (7–10). In those cases, infant formulas aim to mimic human breast milk and its benefits to provide a valuable alternative. Globally, the prevalence of exclusive breastfeeding at 6 months is 48% (11). In Spain, although 90.7% of the mothers initiate breastfeeding, the rate drops to 35.2% at 6 months, similar to other European countries (12, 13).

To narrow the gap with breast milk, infant formulas have evolved significantly since the early 20th century, from simple mixtures of cow’s milk to sophisticated products (14). Recent advances in infant formula research have focused on incorporating bioactive compounds to better mimic human milk composition and functionality. Key additions include Long-chain polyunsaturated fatty acids (LC-PUFA), added to support retinal development, and brain growth/functioning (15) Probiotics and prebiotics are incorporated to modulate gut microbiota and improve gastrointestinal health (16), like human milk oligosaccharides (HMOs), which protect against infections and modulate immune responses (17). Other bioactive compounds like *α*-lactalbumin, nucleotides, and milk fat globule membrane (MFGM) components have shown potential benefits for cognitive development and reducing infections (18–20). Additionally, *β*-casein A2 has emerged as a globally relevant innovation. Unlike A1 *β*-casein, A2 β-casein does not release BCM-7, a peptide linked to digestive discomfort and inflammation. Clinical studies show that milk containing only A2 β-casein improves gastrointestinal tolerance, especially in sensitive populations (21).

When infant formula is necessary, healthcare professionals (HCPs) play a key role in guiding parents toward appropriate options, with pediatricians primarily responsible for prescribing, and pharmacy professionals serving as the main channel for consultation and distribution (22, 23). Parental education, facilitated by HCPs, is crucial in this context, as informed parents are more likely to make appropriate feeding decisions, ensuring safe formula use and reducing the risks of misinformation, especially given the complex landscape of formula options (24). Therefore, given the rapid advances in infant formulas, HCPs must keep up to date with the latest innovations and have a comprehensive understanding of the subject (25).

This study was designed to explore the perceptions, self-perceived knowledge, and questionnaire-based functional recognition of Spanish pediatricians and pharmacy professionals regarding infant formulas, their ingredients, and their biological functions. The survey also aimed to identify differences in self-perceived knowledge, functional recognition, and perceptions between the two groups of healthcare specialists that in Spain are closer to infant formula recommendations: pediatricians and pharmacy professionals.

## 2 Materials and methods

### 2.1 Study design

An observational, cross-sectional, descriptive survey was conducted through social media channels where Spanish healthcare professionals have the most relevant presence (LinkedIn and X/Twitter), and it was also sent to the database of health professionals of Laboratorios Ordesa S. L., a Spanish company specialized in nutrition and food supplements, with a sample of 5,187 HCPs and 2,400 pharmacy professionals who met legal conditions to receive communications and surveys. No specific inclusion criteria were applied. Anonymous and voluntary participation was available from September 2023 to June 2024.

The questionnaire was developed by a team of experts in the field after a comprehensive literature review. It consisted of a total of 28 questions, divided into three sections. The first section focused on sociodemographic characteristics, including age, gender, workplace location (rural as <2,000 inhabitants, semi-urban as 2,000–10,000 inhabitants, or urban as >10,000 inhabitants), and educational background (Table 1).

**Table 1:** Sociodemographic characteristics of the study participants.

Sociodemographic characteristics	Pediatricians (*n* = 505)	Pharmacy professionals (*n* = 371)
Age (years old), mean (SD)	51.7 (10.6)	42.6 (9.7)
Sex, *n* (%)		
Male	204 (40.4)	79 (21.3)
Female	301 (59.6)	292 (78.7)
Workplace location, *n* (%)		
Rural	11 (2.2)	17 (4.6)
Semi-urban	56 (11.3)	84 (22.6)
Urban	429 (86.5)	270 (72.8)
Education, *n* (%)		
Pediatrics	427 (84.6)	N/A
Pediatric gastroenterology	25 (4.9)	N/A
Family doctor working as pediatrician	35 (6.9)	N/A
Other	18 (3.6)	N/A
BSc in pharmacy	N/A	202 (54.5)
BSc in pharmacy and nutrition	N/A	30 (8.1)
BSc in pharmacy and nutritional studies	N/A	21 (5.7)
Pharmacy technician	N/A	47 (12.7)
Assistant pharmacy technician	N/A	71 (19.1)

**Figure 1:**
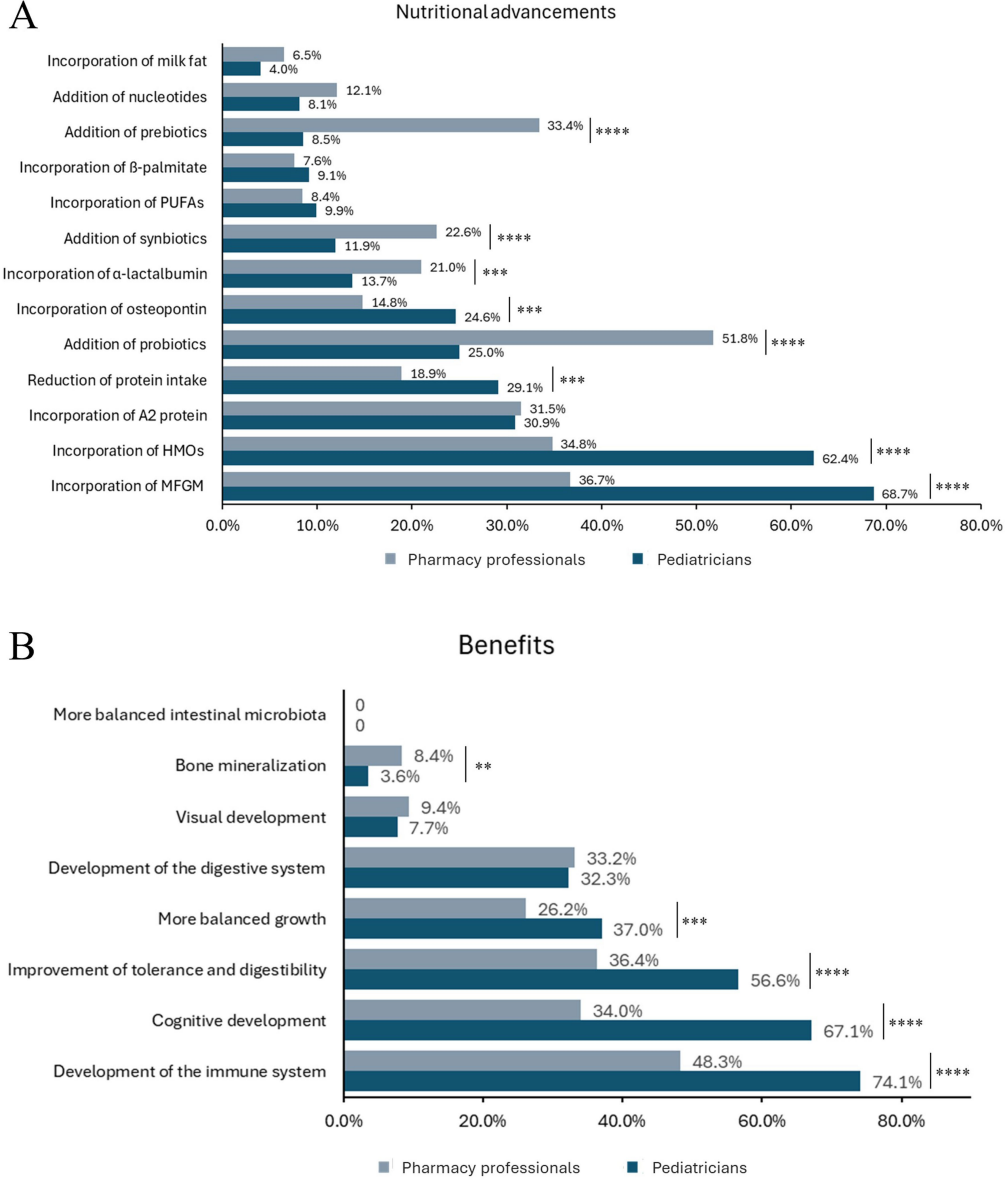
Perceptions about **(A)** the most important nutritional advancements and **(B)** the most valuable benefits of infant formula. Significance was assessed using the Fisher’s exact test. ** *p* < 0.01; *** *p* < 0.001; **** *p* < 0.0001.

The second section assessed participants´ general knowledge and perceptions about infant formulas. The evaluation was performed by rating the degree of agreement from 1 (completely disagree) to 10 (completely agree) of 6 different statements and by answering three closed-ended questions with predefined response options. Closed questions involved most important nutritional advancements, most valuable benefits of infant formulas, and main sources of information (Table 2; Supplementary Table S1; Figure 1).

**Table 2:** Perception and general knowledge about infant formulas.

Statements	Pediatricians Mean (SD)	Pharmacy professionals Mean (SD)	*p*-value
In general terms, I believe I have a solid understanding of infant formulas and the various ingredients/components involved in their formulation.	7.9 (1.3)	6.7 (1.9)	0.0000
I believe it is important to educate parents about the ingredients/composition of infant formulas.	7.8 (1.7)	8.3 (1.9)	0.0000
I believe that infant formulas have evolved significantly over the past 5 years.	9.0 (1.1)	8.3 (1.7)	0.0000
Infant formulas and the innovations in their formulations are a topic that interests me.	8.8 (1.3)	8.4 (1.8)	0.0143
I believe that infant formulas (for healthy infants) are very similar to each other, with few significant differences.	5.0 (2.2)	6.0 (2.3)	0.0000
The more functional ingredients an infant formula contains, the more complete its composition.	7.7 (1.8)	6.9 (2.2)	0.0000

Significance was assessed using the U Mann–Whitney test.

**Figure 2:**
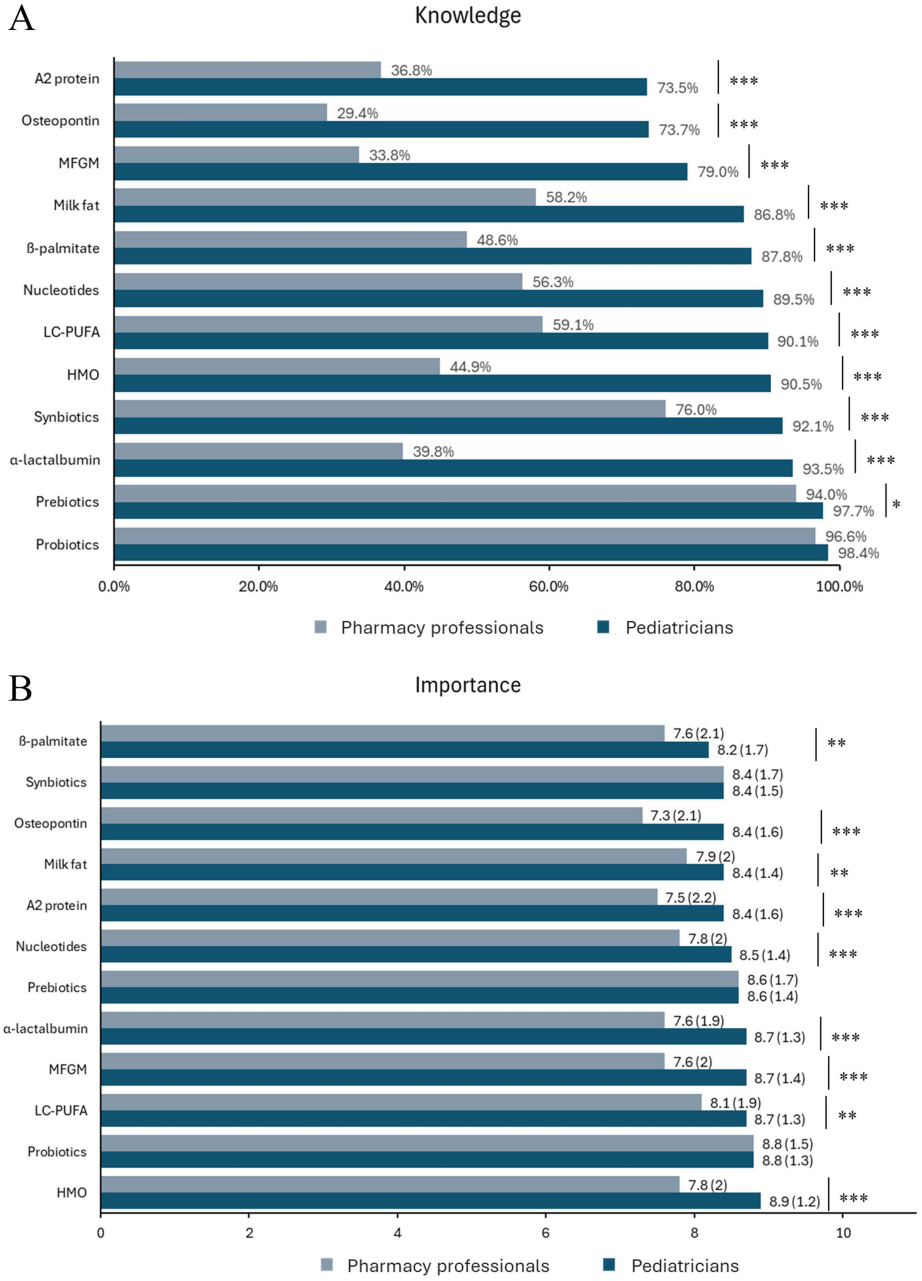
**(A)** Evaluation of participants’ self-perceived knowledge—rated as good or excellent—about the ingredients in infant formula. **(B)** Evaluation of participants’ perception about the importance of ingredients in infant formulas. Perceived importance was evaluated separately on a scale from 1 (*not important at all*) to 10 (*extremely important*). Mean and SD values are shown. Significance was assessed using the Fisher’s exact test. * *p* < 0.05; ** p < 0.01; *** *p* < 0.001.

The third section focused on the participants´ self-perceived knowledge, perceived importance, and functional recognition regarding 12 specific infant formula’s ingredients and their nutritional benefits. Participants’ self-perceived knowledge of each ingredient was assessed using a Likert scale with five response categories: “*I do not know what it is*,” “*It sounds familiar*,” “*Yes, I know it well*” “*Yes, I know it very well*” (Figure 2A). Perceived importance was evaluated separately on a scale from 1 (*not important at all*) to 10 (*extremely important*) (Figure 2B). The success rate was defined as the proportion of participants who correctly identified at least one relevant biological function of each ingredient out of the total number of responses (Supplementary Table S2). This overall success rate was then analyzed in relation to (1) self-perceived knowledge (Table 3), and (2) the degree of agreement with the six statements about infant formulas (Supplementary Table S3).

**Table 3:** Relation of participants’ self-perceived knowledge about the ingredients in infant formula and their success rate in the responses.

Pediatricians	I do not know what it is		It sounds familiar		I know it well		I know it very well		*p*-value
	N*	Success (%)	*N*	Success (%)	*N*	Success (%)	*N*	Success (%)	
MFGM	3	50.0	63	86.3	272	95.1	47	97.9	**0.001**
Milk Osteopontin	3	27.3	42	51.2	217	79.5	38	82.6	**0.001**
α-Lactalbumin	0	–	14	51.9	206	74.4	86	76.1	0.076
A2 Protein	2	28.6	54	71.1	200	82.3	52	82.5	**0.003**
Nucleotides	0	–	20	55.6	215	74.9	62	75.6	**0.038**
HMOs	1	100.0	32	88.9	246	89.8	96	92.3	0.791
Probiotics	0	–	7	100.0	201	96.6	198	98.5	**0.005**
Prebiotics	0	–	8	80.0	243	98.4	163	99.4	**0.005**
Synbiotics	2	100.0	24	85.7	250	98.0	121	98.4	**0.001**
LCPUFAs	0	0.0	21	58.3	219	84.6	88	88.9	**0.001**
Milk Fat	0	0.0	20	44.4	139	49.5	33	43.4	0.364
β-Palmitate	1	100.0	24	51.1	215	77.1	73	85.9	**0.001**

Pharmacy professionals	I do not know what it is		It sounds familiar		I know it well		I know it very well		*p*-value
	N*	% of success	N*	% of success	*N*	% of success	*N*	% of success	
MFGM	14	78.6	93	85.0	78	92.3	9	88.9	0.2553
Milk Osteopontin	12	25.0	92	41.3	65	67.7	2	50.0	**0.0013**
α-Lactalbumin	8	50.0	89	51.7	78	60.3	8	62.5	0.6788
A2 Protein	11	18.2	74	55.4	73	64.4	5	40.0	**0.0239**
Nucleotides	4	0.0	58	56.9	97	60.8	16	81.3	**0.0253**
HMOs	11	45.5	73	80.8	80	91.3	12	83.3	**0.0033**
Probiotics	2	50.0	6	50.0	112	97.3	112	96.4	**0.0002**
Prebiotics	2	50.0	11	72.7	120	96.7	98	96.9	**0.0050**
Synbiotics	3	66.7	26	84.6	104	93.3	56	98.2	**0.0325**
LCPUFAs	3	33.3	42	73.8	72	69.4	30	73.3	0.5146
Milk Fat	0	0	55	45.5	82	34.2	15	26.7	0.2790
β-Palmitate	1	100.0	52	46.2	60	31.7	12	50.0	0.1559

*N value indicates the total number of (correct or incorrect) answers. Significance was assessed using the Fisher’s exact test. Bold values indicate a significant relation between self-perceived knowledge about the ingredients in infant formula and success rate in the responses.

### 2.2 Data analysis

Descriptive statistics were used for quantitative variables (mean and standard deviation), while categorical variables were presented as percentages (%) to describe the sample characteristics. Comparative analyses were conducted with Fisher’s exact test for categorical variables, Mann–Whitney’s test for continuous variables and Kendall’s Tau-b coefficient to assess the correlation between ordinal variables. All statistical analyses were performed using the Statistical Analysis Systems (SAS Institute, Cary, NC, United States) package version 9.4, with a significance level set at *p* < 0.05.

### 2.3 Ethical considerations

The study consisted of an anonymous, voluntary online survey addressed exclusively to HCPs. No patients were involved, no clinical intervention was performed, no medical records were accessed, and no directly or indirectly identifiable personal data or sensitive data were collected. Participation was voluntary, and completion of the questionnaire was considered indicative of willingness to participate.

Data protection requirements were considered in relation to confidentiality and data handling. As no personal data were collected or processed, the study was considered outside the scope of personal data processing under Regulation (EU) 2016/679 and Spanish Organic Law 3/2018 on Personal Data Protection and Digital Rights.

No formal ethics committee approval or waiver was obtained before survey implementation. Ethical approval was not required for this study due to its design and data source that do not involve identifiable personal data or direct intervention in human subjects.

## 3 Results

### 3.1 Sociodemographic data

A total of 876 respondents participated in the survey, including 505 pediatricians and 371 pharmacy professionals (Table 1). The mean age among pediatricians was 51.7 years and 42.6 years among pharmacy professionals. In both groups, the majority of participants were women (59.6 and 78.7%, respectively), and most respondents worked in urban areas (86.5 and 72.8%, respectively). Regarding professional background, physicians specialized in pediatrics (84.6%) were prevalent among the respondents, while the pharmacy group showed more diverse educational profiles, including related-degrees in pharmacy (68.3%) and technician-level qualifications (31.8%).

### 3.2 Self-perceived knowledge and perceptions about infant formulas

When asked about their knowledge and perceptions about infant formulas (Table 2), pediatricians reported a higher self-perceived understanding regarding infant formulas and their components compared to pharmacy professionals (7.9 vs. 6.7; *p* = 0.0000). However, pharmacy professionals expressed stronger agreement with the need to educate parents about the composition and ingredients (7.8 vs. 8.3; *p* = 0.0000). Regarding the development of infant formulas, pediatricians reported a significantly higher level of agreement with both statements “*Iinfant formulas have evolved significantly over the past 5 years*” (9.0 vs. 8.3; *p* < 0.0000), and “*Infant formulas and the innovations in their formulations are a topic that interests me*,” when compared to pharmacy professionals (8.8 vs. 8.4; *p* = 0.0143). Pharmacy professionals were significantly more likely to consider infant formulas as highly similar to one another, with few differences (5.0 vs. 6.0; *p* = 0.0000), and were less likely to agree that a greater number of functional ingredients results in a more complete formula composition (7.7 vs. 6.9; *p* = 0.0000).

### 3.3 Source of information

Indistinctly, both groups identified the pharmaceutical industry (through representatives or training courses) as their primary source of information (Supplementary Table S1). Nonetheless, significant differences emerged among the remaining options. While pediatricians showed a preference for medical conferences (41.4% vs. 6.2%; *p* = 0.0000) and scientific articles (39.8% vs. 23.2%; *p* = 0.0000), pharmacy professionals favored clinical training sessions at healthcare centers (22.4% vs. 38.8%; *p* = 0.0000) or receiving information directly from professional colleagues (5.0% vs. 39.4%; *p* = 0.0000).

### 3.4 Major nutritional advances in infant formulas

Among different nutritional advancements (Figure 1A), pediatricians were significantly more likely to highlight the incorporation of MFGM (*p* < 0.0001), HMOs (*p* < 0.0001), milk osteopontin (*p* < 0.001) and the reduction of protein intake (*p* < 0.001) into infant formulas as the most notable nutritional advances in recent years. In contrast, pharmacy professionals significantly favored the addition of probiotics (*p* < 0.0001), prebiotics (*p* < 0.0001), symbiotics (*p* < 0.0001) and *α*-lactalbumin (*p* < 0.001). When asked to identify the three most valued benefits in infant formulas (Figure 1B), pediatricians more frequently selected immune system development (*p* < 0.0001), cognitive development (*p* < 0.0001), improved digestive tolerance (*p* < 0.0001), and more balanced growth (*p* < 0.001). Pharmacy professionals, in contrast, selected immune system development in first place and improved digestive tolerance in second place.

### 3.5 Infant formulas composition and their relevance

#### 3.5.1 Ingredients

Over 75% of pediatricians reported having good or very good knowledge of all 12 ingredients. A significantly lower level of knowledge was observed among pharmacy professionals for all ingredients (*p* < 0.05) except the probiotics (Figure 2A). In both groups, prebiotics, probiotics, and synbiotics were the ingredients they reported knowing most thoroughly, with pediatricians also indicating a substantially greater awareness of *α*-lactalbumin (93.5% vs. 39.8%; *p* < 0.001). Regarding the importance attributed to each ingredient, pediatricians consistently assigned greater relevance compared to pharmacy professionals in 9 of them (*p* < 0.01) (Figure 2B). Greatest discrepancies were observed for HMOs (8.9 vs. 7.8; *p* < 0.001), LC-PUFAs (8.7 vs. 8.1; *p* < 0.01), MFGM (8.7 vs. 7.6; *p* < 0.001), and α-lactalbumin (8.7 vs. 7.6; *p* < 0.001), while similar levels of importance were attributed to prebiotics, probiotics, and synbiotics.

#### 3.5.2 Ingredients and functions

When participants were asked to match ingredients to their biological functions, pediatricians achieved higher success rates across 11 out of 12 ingredients, with statistically significant differences in 7 of them compared to pharmacy professionals: Milk osteopontin (*p* = 0.0000), *α*-lactalbumin (*p* = 0.0000), A2 protein (*p* = 0.0000), nucleotides (*p* = 0.0026), HMOs (*p* = 0.0258), LC-PUFAs (*p* = 0.0011), and milk fat (*p* = 0.0471) as shown in Table 4. Individualized success rates of the ingredient and its right biological function are shown in Supplementary Table S2.

**Table 4:** Success rate between ingredients and their biological functions.

Ingredients	Pediatricians, *n* (%)	Pharmacy professionals, *n* (%)	*p*-value
MFGM	395 (92.5)	173 (87.4)	0.0512
Milk Osteopontin	304 (72.0)	87 (50.0)	**0.0000**
α-Lactalbumin	313 (73.3)	102 (55.7)	**0.0000**
A2 Protein	320 (78.6)	94 (56.6)	**0.0000**
Nucleotides	306 (72.7)	105 (59.7)	**0.0026**
HMOs	389 (90.3)	148 (83.6)	**0.0258**
Probiotics	424 (97.7)	222 (95.3)	0.1045
Prebiotics	422 (96.8)	220 (94.8)	0.2146
Synbiotics	409 (96.7)	178 (93.2)	0.0573
LCPUFAs	344 (82.5)	107 (69.5)	**0.0011**
Milk Fat	196 (47.0)	58 (37.7)	**0.0471**
β-Palmitate	93 (22.0)	50 (39.7)	**0.0001**

Success rate was defined as the proportion of participants who correctly identified at least one relevant biological function for each ingredient. Significance was assessed using the Fisher’s exact test. Bold values indicate a significant difference between pediatricians and pharmacy professionals in identifying ingredients biological functions.

In addition, the degree of success was evaluated according to the level of self-reported knowledge in both groups (Table 3). Among pediatricians, the success rate increased steadily with higher levels of perceived knowledge, reaching statistical significance for several ingredients. For MFGM, correct identification rose from 50.0% among those unfamiliar with the ingredient to 97.9% among those reporting very good knowledge (*p* = 0.001). A similar pattern was observed for milk osteopontin (27.3 to 82.6%; *p* = 0.001), and A2-protein (28.6 to 82.5%; *p* = 0.003). Notably, pediatricians who reported knowing an ingredient well or very well achieved accuracy rates ≥90% in five of the 12 components assessed (MFGM, HMOs, probiotics, prebiotics, and synbiotics). In most cases, even when differences were not statistically significant, accuracy still remained high among those declaring greater familiarity. The exception was milk fat, where correct identification did not improve substantially despite higher self-perceived knowledge.

Among pharmacy professionals, similar significant trends were observed for nucleotides (0.0 to 81.3%; *p* = 0.025), HMO (45.5 to 83.3%; *p* = 0.003), probiotics (50.0 to 96.4%; *p* = 0.0002), prebiotics (50.0 to 96.9%; *p* = 0.005), and synbiotics (66.7 to 98.2%; *p* = 0.033). Pharmacy professionals who reported knowing an ingredient well or very well achieved accuracy rates ≥90% in three of the 12 components assessed (probiotics, prebiotics, and synbiotics). In contrast, MFGM and milk osteopontin did not reach statistical significance in this group, despite showing an upward trend in accuracy across knowledge levels. Ingredients showing consistent patterns of higher success related to better knowledge in both groups were prebiotics, probiotics, synbiotics, MFGM, and HMO.

Finally, the success rate was correlated with the degree of agreement of the six statements about infant formulas (Supplementary Table S3). Among pediatricians, a significant positive correlation was observed between those with higher success rates and those who strongly agreed that infant formulas with more functional ingredients are more complete (KT-b = 0.087; *p* = 0.018) and that it is important to educate parents about them (KT-b = 0.084, *p* = 0.022). On the other hand, a significant negative correlation (KT-b = −0.073) was observed between success rate and agreement with the statement “*I believe that infant formulas (for healthy infants) are very similar to each other, with few significant differences*” (*p* = 0.043), a pattern which was also observed among pharmacy professionals (*p* = 0.0051). Finally, pharmacy professionals with a strong interest in infant formulas and their innovations were significantly associated with a higher success rate (KT-b = 0.28026; *p* = 0.0015).

## 4 Discussion

Infant formula composition has evolved rapidly in response to emerging science around bioactive ingredients. While pediatricians and pharmacy professionals play complementary roles in advising caregivers, their respective knowledge, perceptions, and engagement regarding formula ingredients vary considerably.

Our data revealed that responding Spanish pediatricians reported significantly higher self-perceived knowledge of infant formula ingredients compared to responding Spanish pharmacy professionals. This aligns with their greater expressed interest in the topic, stronger recognition of the rapid advancements in formula composition over the past 5 years, and superior questionnaire-based performance in identifying at least one relevant biological function of 7 out of 12 key ingredients, when compared to responding pharmacy professionals. Surveyed pediatricians also more strongly rejected the claim that infant formulas for healthy infants are very similar and have few significant differences, suggesting greater awareness of product variability and ongoing innovation in formula science, likely due to their more frequent clinical involvement in infant nutrition decisions (22, 23).

Interestingly, this population of pharmacy professionals expressed stronger agreement than surveyed pediatricians on the importance of educating parents about formula composition. This may reflect their frequent direct contact with caregivers. A study by Tsuyuki et al. reported that in the United States, patients visit pharmacists approximately 30 times per year, compared to only 2.9 visits to family physicians. Similarly, in a Canadian cohort of patients with chronic conditions, patients saw pharmacists more than twice as often as general practitioners (15 vs. 7 visits annually) (26). While these figures are from North American settings, were access to medical consultations have a lower accessibility and higher price than in our setting, our findings may suggest a similar dynamic as well: while pediatricians are more involved in the clinical decision-making process and show a deeper understanding of formula composition, pharmacies are a key access point for parents and caregivers at the time of formula purchase, and pharmacy professionals are frequently consulted for practical advice in a highly accessible setting. This day-to-day proximity may increase their perceived responsibility for caregiver education, even when technical performance in ingredient function identification is lower. This division of roles highlights the importance of establishing coordinated communication strategies to ensure that families receive consistent, evidence-based information from both prescribers and dispensers.

When examining the relationship between self-reported knowledge and questionnaire-based performance in identifying at least one relevant biological function of formula ingredients, this study’s data showed a generally positive association: higher self-perceived knowledge tended to align with greater accuracy. However, this pattern varied by ingredient. Notably, both responding pediatricians and pharmacy professionals who rated themselves as highly knowledgeable demonstrated lower accuracy when asked about the functions of specific ingredients such as milk osteopontin, *α*-lactalbumin, A2 protein, milk fat, and *β*-palmitate. These ingredients were also rated as less important overall, suggesting that unfamiliarity may lead to underestimation of their clinical value, even among surveyed professionals who consider themselves well-informed.

This misalignment becomes even more evident when considering the relationship between the most highly valued formula benefits and the ingredients that were least accurately identified. A negative correlation appears to emerge: the greater the perceived benefit of the formula, the lower the accuracy in identifying the ingredients responsible for it. For example, immune system development was consistently rated as the most important benefit, yet key immune-supporting ingredients such as milk osteopontin were among the least correctly identified. Similarly, improved digestibility was another top-rated benefit, with A2 protein and milk fat nutrients showing lower success rates in function identification. The same pattern was observed for bone mineralization, supported by ingredients like milk fat and *β*-palmitate. These findings suggest a relevant discordance among the surveyed population: while professionals recognize the clinical relevance of certain outcomes, their questionnaire-based ability to link these outcomes with the corresponding ingredients was variable.

Similar inconsistencies between self-perceived and questionnaire-based knowledge assessments have been documented in other areas of pediatric healthcare, such as palliative care and neonatal jaundice (27, 28). One likely explanation for this discordance between perceived familiarity and questionnaire-based functional recognition is the rapid pace of change in formula science. New ingredients like HMOs, MFGM, and A2 protein are being introduced regularly, each with complex, multi-system functions. These compounds often impact immune health, gut function, neurodevelopment, and metabolism simultaneously, making it difficult for professionals to stay current. Also, this biological complexity, combined with a lack of standardized education around dosage creates an environment where even engaged professionals may struggle to stay up to date (14, 19, 29, 30). This point is especially relevant for pharmacy professionals, due to the high number of different formulations and categories that are involved in their daily activity and the large number of new product launches, which makes it complicated to maintain an updated knowledge of all the latest developments and remember the composition of existing preparations.

Another factor influencing professional knowledge may be the nature of information sources available for the studied professionals. The high reliance on industry-provided information observed in both professional groups should be interpreted not as a bias introduced by the study, but as a reflection of the current information ecosystem through which many professionals receive updates in infant nutrition. Industry-provided information may partly explain why widely marketed ingredients or those included in a larger number of formulas (such as probiotics and prebiotics) were more readily recognized, whereas newer or less widely implemented components (such as milk osteopontin or A2 protein) showed lower accuracy rates. This pattern supports the need to strengthen access to independent educational resources and standardized curricula support to ensure more balanced, evidence-based knowledge acquisition across both prescribers and dispensers.

To our knowledge, this is one of the first studies to examine, at ingredient level, HCPs’ self-perceived knowledge, perceived importance, and ability to identify at least one relevant biological function of specific infant formula components. Our findings suggest potential opportunities for targeted strategies to strengthen both foundational knowledge and communication practices across disciplines. Potential actions include (1) continued education initiatives incorporating updated, ingredient-specific modules with clear links between nutritional compounds and clinical outcomes, delivered through accredited courses, webinars, or professional society guidelines and (2) development of digital decision-support tools, such as ingredient comparison apps or function-based labeling aids at the point of care; may bridge knowledge gaps, ensure consistent communication to caregivers, and may help professionals provide more accurate advice in real-time. In addition, these initiatives should be tailored to professional training profiles and scopes of practice; in particular, pharmacy technicians may benefit from dedicated modules and simplified educational resources focused on the ingredient–function relationship, complemented by clear counseling algorithms appropriate for point-of-dispensing interactions. Greater collaboration between pediatricians and pharmacy professionals through joint training programs or interdisciplinary workshops may also promote aligned messaging to caregivers.

This study has several limitations. First, the recruitment strategy may have introduced selection bias. The survey was disseminated through professional social media channels and through a professional database, and participation was voluntary. Therefore, respondents may have had greater baseline interest in infant nutrition, greater familiarity with infant formula composition, or previous exposure to educational activities on this topic than non-respondents. As a result, the findings should not be interpreted as representative of all Spanish pediatricians or pharmacy professionals, but rather as descriptive of the professionals who responded to the survey. In addition, because part of the recruitment was conducted through open social media dissemination, an exact overall response rate could not be calculated. As a contextual estimate, the number of respondents corresponded to approximately 11.5% of the total professional database used for e-mail dissemination (876 respondents out of 7,587 professionals). However, this figure should not be interpreted as a formal response rate because the recruitment source of each respondent was not tracked and the denominator for social media dissemination was unknown. Therefore, the possibility of non-response bias should therefore be considered, as professionals who chose to participate in an infant formula-related survey may differ systematically from those who did not.

Second, the questionnaire was developed by the study team after a literature review but was not subjected to formal psychometric validation. No pilot testing, cognitive interviewing, internal consistency assessment or test–retest reliability analysis was performed. Therefore, the results related to perceived knowledge, perceptions and functional recognition should be interpreted with caution. In particular, the observed differences between self-perceived knowledge and correct identification of biological functions may partly reflect limitations of the measurement instrument rather than only true discrepancies in professional knowledge.

Third, the success rate was defined as the correct identification of at least one relevant biological function for each ingredient. This criterion was intended to capture basic functional recognition, but it does not provide a comprehensive measure of ingredient-specific knowledge and may overestimate actual performance, particularly if participants selected both correct and incorrect options. Future studies should consider more stringent scoring systems, including identification of the primary function, penalization of incorrect responses, or validated composite knowledge scores.

Finally, the sample may not fully capture professionals working in rural or underserved settings, where access to continuing education and exposure to newer infant formula ingredients may differ. Despite these limitations, the study included a large sample of pediatricians and pharmacy professionals and provides an ingredient-specific exploratory assessment of self-perceived knowledge, perceived importance, and questionnaire-based functional recognition of infant formula components.

## 5 Conclusion

In conclusion, this study highlights the complementary differences between responding pediatricians and pharmacy professionals in their approach to infant nutrition. In this questionnaire, pediatricians demonstrated greater ability to identify at least one relevant biological function of several infant formula ingredients, while pharmacy professionals stood out for their proximity to families and their involvement in parent education. The variations in self-perceived knowledge and functional recognition identified between the groups should be interpreted as potential opportunities to strengthen ongoing professional development and foster collaborative work, in line with scientific advances in infant nutrition. By harmonizing knowledge with coordinated communication, both profiles may contribute to informed, consistent, and evidence-based guidance for caregivers.

## Data Availability

The original contributions presented in the study are included in the article/Supplementary material, further inquiries can be directed to the corresponding author/s.

## References

[1] Dimitroglou M Iliodromiti Z Christou E Volaki P Petropoulou C Sokou R . Human breast milk: the key role in the maturation of immune, gastrointestinal and central nervous systems: a narrative review. Diagnostics (Basel). (2022) 12. doi: 10.3390/diagnostics12092208, 36140609 PMC9498242

[2] Zhang M Qiao H Yang S Kwok L-Y Zhang H Zhang W. Human breast milk: the role of its microbiota and metabolites in infant health. J Agric Food Chem. (2024) 72:10665–78. doi: 10.1021/acs.jafc.3c07690, 38691667

[3] Katiyar K Singh S Yadav S Verma B Dr. Breast milk vs. infant formula: a comprehensive review of nutritional content, health outcomes, and practical considerations. Int J Sci Res. (2024) 1:16–20. doi: 10.36106/ijsr/5303875

[4] Nuzzi GTI DI Cicco ME Peroni DG. Breast milk: more than just nutrition! Minerva Pediatr. (2021) 73:111–4. doi: 10.23736/s2724-5276.21.06223-x 33880902

[5] Meek JY Noble L Section on B. Policy statement: breastfeeding and the use of human milk. Pediatrics. (2022) 150:988. doi: 10.1542/peds.2022-057988, 35921640

[6] Perez-Escamilla R Buccini GS Segura-Perez S Piwoz E. Perspective: should exclusive breastfeeding still be recommended for 6 months? Adv Nutr. (2019) 10:931–43. doi: 10.1093/advances/nmz039, 31147672 PMC6855974

[7] Johnston L. Infant formulas explained. S Afr Fam Pract. (2014) 53:433–6. doi: 10.1080/20786204.2011.10874128, 37339054

[8] Kozhimannil KB Jou J Attanasio LB Joarnt LK McGovern P. Medically complex pregnancies and early breastfeeding behaviors: a retrospective analysis. PLoS One. (2014) 9:e104820. doi: 10.1371/journal.pone.0104820, 25118976 PMC4132072

[9] Martin CR Ling PR Blackburn GL. Review of infant feeding: key features of breast Milk and infant formula. Nutrients. (2016) 8:279. doi: 10.3390/nu8050279, 27187450 PMC4882692

[10] Pina-Perez MC Martinez A Rodrigo D. New advances in infant feeding: new products and novel technologies. Recent Pat Food Nutr Agric. (2017) 8:152–65. doi: 10.2174/2212798409666170328145150, 28355995

[11] UNICEF. *Rates of Breastfeeding Increase around the World through Improved Protection and Support*. (2023). Available online at: https://www.unicef.org/media/150586/file/Global (Accessed Semptember, 2025).

[12] Martin-Ramos S Dominguez-Aurrecoechea B Garcia Vera C Lorente García Mauriño AM Sánchez Almeida E Solís-Sánchez G. Lactancia materna en España y factores relacionados con su instauración y mantenimiento: estudio LAyDI (PAPenRed). Aten Primaria. (2024) 56:102772. doi: 10.1016/j.aprim.2023.102772, 37741187 PMC10520303

[13] Theurich MA Davanzo R Busck-Rasmussen M Díaz‐Gómez NM Brennan C Kylberg E . Breastfeeding rates and programs in Europe: a survey of 11 national breastfeeding committees and representatives. J Pediatr Gastroenterol Nutr. (2019) 68:400–7. doi: 10.1097/MPG.0000000000002234, 30562307

[14] Hernell O. Human milk vs. cow's milk and the evolution of infant formulas. Nestle Nutr Workshop Ser Pediatr Program. (2011) 67:17–28. doi: 10.1159/000325572, 21335987

[15] Zou L Pande G Akoh CC. Infant formula fat analogs and human milk fat: new focus on infant developmental needs. Annu Rev Food Sci Technol. (2016) 7:139–65. doi: 10.1146/annurev-food-041715-033120, 26934172

[16] Bakshi S Paswan VK Yadav SP Bhinchhar BK Kharkwal S Rose H . A comprehensive review on infant formula: nutritional and functional constituents, recent trends in processing and its impact on infants' gut microbiota. Front Nutr. (2023) 10:101194679. doi: 10.3389/fnut.2023.1194679, 37415910 PMC10320619

[17] Cheng YJ Yeung CY. Recent advance in infant nutrition: human milk oligosaccharides. Pediatr Neonatol. (2021) 62:347–53. doi: 10.1016/j.pedneo.2020.12.013, 33893051

[18] Almeida CC Mendonca Pereira BF Leandro KC Costa MP Spisso BF Conte-Junior CA. Bioactive compounds in infant formula and their effects on infant nutrition and health: a systematic literature review. Int J Food Sci. (2021) 2021:1–31. doi: 10.1155/2021/8850080, 34095293 PMC8140835

[19] De Almagro Garcia MC Moreno Munoz JA Jimenez Lopez J. Nuevos ingredientes en fórmulas infantiles. Beneficios sanitarios y funcionales. Nutr Hosp. (2017) 34:8–12. doi: 10.20960/nh.1564, 29156925

[20] Skolnick J Chou C Miklavcic J. Insights into novel infant milk formula bioactives. Nutr Diet Suppl. (2020) 12:11–9. doi: 10.2147/nds.S192099

[21] Jianqin S Leiming X Lu X Yelland GW Ni J Clarke AJ. Effects of milk containing only A2 beta casein versus milk containing both A1 and A2 beta casein proteins on gastrointestinal physiology, symptoms of discomfort, and cognitive behavior of people with self-reported intolerance to traditional cows' milk. Nutr J. (2016) 15:35. doi: 10.1186/s12937-016-0147-z, 27039383 PMC4818854

[22] Fewtrell M Bandsma RHJ Baur L Duggan CP Dumrongwongsiri O Hojsak I . Role of pediatricians in promoting and supporting breastfeeding: a position paper of the international pediatric association strategic advisory group on infant, child, and adolescent nutrition. Ann Nutr Metab. (2023) 79:469–75. doi: 10.1159/000534004 37673040

[23] Smith JD Clinard V Barnes CL. Pharmacists’ guide to infant formulas for term infants. J Am Pharm Assoc. (2003) 51:e28–37. doi: 10.1331/JAPhA.2011.09125 21555278

[24] Malek L Fowler H Duffy G Katzer L. Informed choice or guessing game? Understanding caregivers' perceptions and use of infant formula labelling. Public Health Nutr. (2019) 22:273–86. doi: 10.1017/S1368980018003178, 30477595 PMC10260669

[25] Hvatum I Glavin K Irjall M Cand san AME Holmberg Fagerlund B. Health professionals' counselling on the use of infant formula: a scoping review. Public Health Nurs. (2024) 41:1224–33. doi: 10.1111/phn.13355, 38973632

[26] Tsuyuki RT Beahm NP Okada H al Hamarneh YN. Pharmacists as accessible primary health care providers: review of the evidence. Can Pharm J (Ott). (2018) 151:4–5. doi: 10.1177/1715163517745517, 29317929 PMC5755826

[27] Dotan M Kovalski Y Osovsky D Levine H Osovsky M. Pediatricians’ knowledge and perception of the care of the infant with jaundice. J Pediatr Neonatal Individ Med. (2025) 14:e140111. doi: 10.7363/140111

[28] Zanello E Vecchi R Zamagni G Biagi MC Bruno I Cragnolin E . Measuring perceived, wished and actual knowledge of healthcare providers about pediatric palliative care: development and validation of an online questionnaire in Italy (2023) 11:1971. doi: 10.21203/rs.3.rs-2441773/v1, 37444805 PMC10341144

[29] Karasalih B Duman H Bechelany M Karav S. Osteopontin: its properties, recent studies, and potential applications. Int J Mol Sci. (2025) 26:868. doi: 10.3390/ijms26125868, 40565331 PMC12192592

[30] Rodrigo ML Tymann HA Lochen HA Shores DR. Infant formula ingredients: updates for clinicians. J Pediatr Gastroenterol Nutr. (2024) 78:1005–8. doi: 10.1002/jpn3.12192, 38529854

