# Supplementary material for: Healthcare professionals’ knowledge and perceptions of infant formulas composition: a comparative study of Spanish pediatricians and pharmacy professionals
Source: Front Nutr. 2026 Jul 8;13:1801406. doi: 10.3389/fnut.2026.1801406 (PMC13390703; doi:10.3389/fnut.2026.1801406)
Supplement: Supplementary file 1 [file Data_Sheet_1.PDF]

Supplementary Material

1 Supplementary Data

Table S1. Sources of information about infant formulas.

**What are your 2 main sources of information regarding the ingredients/composition of infant formulas?**			
	**Pediatricians, n (%)**	**Pharmacy professionals, n (%)**	**p-value**
**Pharmaceutical industry (representatives, training/workshop courses)**	445 (88.1)	321 (86.5)	0.5360
**Medical conferences**	209 (41.4)	23 (6.2)	**0.0000**
**Scientific journals**	201 (39.8)	86 (23.2)	**0.0000**
**Training and clinical sessions in the hospital/health center**	113 (22.4)	144 (38.8)	**0.0000**
**Information received through other professional colleagues**	25 (5.0)	146 (39.4)	**0.0000**

Significance was assessed using the Fisher's exact test

Table S2. Success rate in the relation between ingredients and their biological function

	Cognitive development	Visual development	Digestive system development	Immune system development	More balanced growth	Bone mineralization	Improved tolerance and digestibility	More balanced gut microbiota
**MFGM**[1]	**80.6 %**<br>*75.3%*<br>p=0.1408			**64.4 %**<br>*57.6 %*<br>p=0.0926				
**Milk Osteopontin**[2]				**72.0 %**<br>*50.0 %*<br>p=0.0000				
**Alpha-lactalbumin**[3]				**53.6 %**<br>*40.4 %*<br>p=0.0035	**40.1 %**<br>*29.5 %*<br>p=0.0136			
**A2 protein**[4]							**78.6 %**<br>*56.6 %*<br>p=0.0000	
**Nucleotides**[5]			**47.5 %**<br>*30.7 %*<br>p=0.0001	**66.0 %**<br>*51.7 %*<br>p=0.0012				
**HMO**[6]			**42.5 %**<br>*33.9 %*<br>p=0.0557	**66.4 %**<br>*43.5 %*<br>p=0.0000				**62.0 %**<br>*61.0 %*<br>p=0.8545
**Probiotics**[7]			**56.0 %**<br>*60.9 %*<br>p=0.2498	**59.2 %**<br>*58.4 %*<br>p=0.8690				**90.3 %**<br>*85.4 %*<br>p=0.0729
**Prebiotics**[8]			**52.5 %**<br>*55.2 %*<br>p=0.5160	**52.3 %**<br>*43.5 %*<br>p=0.0346				**87.6 %**<br>*84.1 %*<br>p=0.2360
**Synbiotics**[9]			**53.5 %**<br>*55.0 %*<br>p=0.7267	**55.6 %**<br>*57.6 %*<br>p=0.6611				**87.2 %**<br>*74.4 %*<br>p=0.002
**LC-PUFA**[10]	**77.9 %**<br>*61.7 %*<br>p=0.0002	**67.9 %**<br>*55.8 %*<br>p=0.0102						
**Milk fat**[11]						**13.9 %**<br>*24.0 %*<br>p=0.0053	**41.0 %**<br>*18.2 %*<br>p=0.0000	
**ß-palmitate**[12]						**35.5 %**<br>*26.0 %*<br>p=0.0016	**59.8 %**<br>*26.2 %*<br>p=0.0000	

Bold: pediatrician´s responses. *Italics*: pharmacy professionals´ responses. Significance was assessed using the Fisher's exact test.

Table S3. Correlation between the statements and their success rate in the responses.

	**Pediatricians**		**Pharmacy professionals**	
	**Kendall´s Tau-b**	**p-value**	**Kendall´s Tau-b**	**p-value**
**In general terms, I believe I have a solid understanding of infant formulas and the various ingredients/components involved in their formulation.**	0.057	0.123	0.09340	0.2737
**I believe that infant formulas have evolved significantly over the past 5 years.**	0.071	0.062	0.14012	0.1087
**Infant formulas and the innovations in their formulations are a topic that interests me.**	0.031	0.408	0.28026*	**0.0015**
**I believe that infant formulas (for healthy infants) are very similar to each other, with few significant differences.**	-0.073*	**0.043**	-0.23262	**0.0051**
**The more functional ingredients an infant formula contains, the more complete its composition.**	0.087*	**0.018**	-0.06203	0.4607
**I believe it is important to educate parents about the ingredients/composition of infant formulas.**	0.084*	**0.022**	0.16486	0.0588

*Low correlation.

Significance was assessed using the Tau Kendall’s test
